# Leveraging Responsive Feedback to Redesign a Demand Generation Strategy: Experience From the IntegratE Project in Lagos State, Nigeria

**DOI:** 10.9745/GHSP-D-22-00244

**Published:** 2023-12-18

**Authors:** Emeka Emmanuel Okafor, Bolaji Gbenuade Oladejo, Michael Alagbile, Sikiru Baruwa, David O. Ayinde, Jennifer Anyanti, Toyin O. Akomolafe

**Affiliations:** a IntegratE Project, Society for Family Health Nigeria, Abuja, Nigeria.; b Population Council, Abuja, Nigeria.; c Society for Family Health, Abuja, Nigeria.

## Abstract

Use of modern contraceptives in Nigeria is low (12%) despite very high knowledge of modern methods (92%). Knowledge of modern methods of contraception in Lagos and Kaduna is high at nearly 100% and 94%, respectively, yet contraceptive use in Lagos and Kaduna remains low at 29% and 14%, respectively. Demand generation was a key strategy in increasing contraceptive uptake through the involvement of providers in the private sector (community pharmacists and patent and proprietary medicine vendors) in family planning service delivery.

We describe how an interpersonal communication strategy was modified using a responsive feedback approach and detail the insights gained in implementing an individual compared with a group interpersonal communication strategy during the implementation of the IntegratE Project in Lagos State, Nigeria. We conducted monthly pause-and-reflect sessions for interpersonal communication agents to pause and reflect on lessons learned and address challenges. Using the responsive feedback approach helped to improve contraceptive uptake by adopting a group strategy. Also, the monthly pause-and-reflect sessions have helped interpersonal communication agents see program implementation as a collective responsibility.

## Abstract (precis)

Authors detail using a responsive feedback approach to reflect on successes and challenges of an interpersonal communication agent strategy and adapt the strategy to address gaps in performance to increase contraceptive uptake.

## Teaser

**Key Findings:**

- Using pause-and-reflect sessions promoted learning and allowed project implementers to course correct during activity implementation, resulting in the adaption of the interpersonal communication strategy to better fit the local context.
- Monthly pause-and-reflect sessions helped build interpersonal communication agents' confidence and improve coordination in contributing toward their shared goal of improving contraceptive method uptake.
- Adaptation of the individual interpersonal communication agent strategy to a group strategy contributed to the development of a more efficient working relationship between trained providers, interpersonal communication agents, and community gatekeepers, thereby reducing missed opportunities for family planning services for clients referred.

**Key Implications:**

- Project leadership should consider incorporating pause-and-reflect sessions into program design and implementation to facilitate learning that can improve program outcomes.
- Pause-and-reflect sessions should engage both project staff and external stakeholders to ensure collaboration and group reflection on strengthening project implementation.

## INTRODUCTION

Low demand for family planning (FP) services contributed to a low contraceptive prevalence rate in Nigeria. Despite very high knowledge of modern methods (92%), use of modern contraceptives in Nigeria remains low (12%). Similar trends are found in most states in Nigeria. Knowledge of modern methods of contraception in Lagos and Kaduna is high, nearly 100% and 94%, respectively, yet contraceptive use in Lagos and Kaduna remains low, 29% and 14%, respectively.^1^ Access to modern contraceptive methods plays a significant role in increasing contraceptive uptake.^2^ In Nigeria, the private sector plays a substantial role in providing modern contraceptives to users. Although the public sector remains the primary source, with a share of 54%, 41% of modern contraceptive users got their most recent modern contraceptive method from the private sector. Within the private sector, 12% of users acquired contraceptives from community pharmacists (CPs), 22% obtained them from patent and proprietary medicine vendors (PPMVs), 5% from private hospitals/clinics, and the remaining 2% obtained them from other private medical sector providers.^1^ These private-sector suppliers have been identified for their relevance in bridging the gap to increasing access to FP services.

Demand generation approaches have been used in many countries to increase the use of FP service delivery points.^3^^–^^5^ These activities are some of the best practices known to contribute to increased uptake of modern contraceptive methods^3^ and are essential to provide information on FP, address myths and misinformation, increase awareness, and generate discussion about contraceptives.^3^^,^^4^ However, not all demand generation activities translate into use when compared with high knowledge of contraceptives. For example, a study conducted in Ethiopia showed that 74% of women exposed to mass media in urban areas did not use modern contraceptives.^6^

Building a feedback loop into demand generation activities may produce actionable feedback that may reduce wasted efforts and improve the success of such interventions. By continuously gathering feedback through pause-and-reflect sessions, organizations can better understand the needs and preferences of their audience, tailor their strategies accordingly, and ensure that their demand generation activities align well with the intended goals. Social and behavioral interventions, like demand generation interventions, are influenced by several internal and external factors, such as cultural, socioeconomic, organizational, and geographical factors, that determine their outcomes.^6^^,^^7^ Introduction of forums or avenues for learning, feedback, and modifications may improve the effectiveness of social and behavioral interventions.^7^ Interpersonal communication (IPC), a key demand generation strategy adopted by the IntegratE project, involves home visits by peer educators or community mobilizers to provide FP information and counseling, promote long-acting methods, and provide referrals. It is an important strategy for encouraging FP use through one-on-one interactions.^4^^,^^5^ However, evidence indicates that further efforts are required to enhance the effectiveness of the IPC strategy in achieving increased contraceptive use.^5^

The IntegratE project adopted an interpersonal communication strategy as a demand generation activity to encourage contraceptive uptake.

We describe how an IPC strategy implemented by the IntegratE Project in Lagos State, Nigeria, was modified using a responsive feedback approach and compare the results of an individual IPC strategy with an adopted group IPC strategy. Pause-and-reflect sessions were conducted to engage key stakeholders, including implementing teams, and program participants. The session aimed to gather valuable insights, perspectives, and recommendations on the IPC activities and appropriate demand creation approach that would inform the ongoing iteration and refinement of interventions. By actively seeking feedback from these diverse stakeholders, the pause-and-reflect sessions provided a platform for open dialogue and collaboration. We also provide key lessons learned that may be useful for designing a demand generation strategy.

## INTEGRATE PROJECT DESCRIPTION

The IntegratE Project, funded by the Bill & Melinda Gates Foundation and MSD for Mothers, was implemented from 2017 to 2021 to increase access to contraceptive methods through the involvement of private sector providers (CPs and PPMVs) in FP service delivery in Lagos and Kaduna States. In collaboration with stakeholders, the project built the capacity of CPs and PPMVs to provide quality and expanded FP services. The project goal was to meet the modern contraceptive needs of 1,759,577 clients (both new and repeat clients).

CPs and PPMVs were not traditional outlets for contraceptive methods, especially the long-acting reversible methods. Therefore, it was very important to create awareness within the communities of the provision of FP services by these informal providers and generate demand for service uptake through them. The demand generation strategy used by the project included an online communications campaign, mobile phone technology in marketing (sponsored health advertising messages, short videos, and newsletters), and community interpersonal communication agents (IPCAs). The IntegratE project engaged IPCAs to create awareness and increase demand for FP services among women of childbearing age and referred them to CPs and PPMVs' stores that provided FP services. The intervention encompassed both men and women as its target audience, with a particular emphasis on women as the primary focus. Although the primary focus of the intervention was on FP, it is worth noting that women and children were also referred for integrated community case management of childhood illnesses. This expanded scope ensured that not only FP needs were addressed but also the health care requirements of women and children, particularly in the context of managing and treating common childhood illnesses. IPCAs also provided real-time feedback on a regular basis to the project team to improve performance. The community IPCA approach involved in-person interactions with clients at the household, community, and facility levels through individual and group sessions.

IPCAs that met set criteria (e.g., lived within the communities, were young adults, and had previous experience volunteering as IPCAs) were recruited from implementing communities in both states. During the commencement of the project in Ikorodu, Lagos State, 4 IPCAs were initially enlisted. However, as the IPCA strategy underwent modifications, an additional 2 IPCAs were subsequently engaged to strengthen the team. At a later stage, 1 IPCA was discontinued without a replacement. IPCAs' ages ranged between 25 and 35 years, and all of them had at least a secondary school education. Among the IPCAs, 4 were married and 5 were female.

The IPCAs underwent a comprehensive training that spanned 3 days in a classroom setting. Additionally, 1 day was dedicated to practical fieldwork, allowing them to apply the knowledge and skills acquired during the training. IPCAs were trained to conduct individual demand generation activities by providing FP messages and basic counseling using detailed flipcharts. The project decided that IPC mobilization should be done individually because it was assumed that the individual strategy encouraged privacy and confidentiality, which are essential when providing FP counseling. IPCAs visited homes separately to provide information about FP services and referred interested community members to trained CPs and PPMVs within a mapped community using a referral slip. The IPC session was considered complete when clients presented the referral card to a CP or PPMV for contraceptive uptake. The IPC supervisor retrieved the referral cards/slips from the CPs and PPMVs at the end of the month as feedback that the referral process was completed and whether the IPC session resulted in contraceptive method uptake.

Data on IPC sessions, both complete and incomplete, were collated and analyzed monthly to monitor the performance of the demand generation strategy. The routine monthly data findings were discussed during monthly pause-and-reflect sessions held between IPC supervisors and IPCAs to ensure actions were promptly taken to address the issues identified.

## PROJECT MANAGEMENT USING A RESPONSIVE FEEDBACK APPROACH

The IntegratE Project implemented pause-and-reflect sessions, such as quarterly review meetings for CPs and PPMVs and monthly review meetings of IPCAs, to monitor and address issues related to access and utilization of FP service delivery points, provide feedback, and develop action points to improve performance of IPCAs, and ensure accountability. Review meetings or pause-and-reflect sessions are a peer-to-peer knowledge-sharing and learning approach to discuss lessons learned, successes, and challenges and provide feedback for program implementers to adapt implementation.^8^ Many health programs, especially immunization programs, adopted review meetings among peers as a way of improving performance.^8^ IPCA monthly pause-and-reflect sessions served as a support forum for IPCAs to review mobilization data, discuss challenges with mobilization activities, and proffer solutions.

IPCA monthly pause-and-reflect sessions were held on the last working day of the month and occasionally on the first working day of the month for about 4 hours. Meetings were held in person, and IPC supervisors ensured the participation of all IPCAs by notifying them and providing them with a transport stipend.

Pause and reflect is a practice of ongoing learning and adaptation that involves gathering information through monitoring and evaluation, research, or experience; reflecting on information; determining implications; and acting on decisions reached for continuous improvement.^9^^,^^10^ The monthly pause-and-reflect sessions were designed as a combination of an after-action review, which facilitates reflecting on the activities undertaken in the previous month, assessing achievements, and identifying areas for improvement, and a data review, which involves analyzing performance trends. These sessions provide an opportunity to evaluate past actions, learn from experiences, and use data-driven insights to inform future decision-making and enhance overall performance. IPCA pause-and-reflect sessions usually facilitated by the IPC supervisor sought to provide continuous feedback to IPCAs, identify matters arising, and develop action points that would have translated referrals to more women accessing FP services from CPs and PPMVs.

Specifically, IPCAs were asked the following questions: (1) What did we set out to do (i.e., total number of one-on-one mobilization activities planned for the month and what was achieved)? (2) What went well during IPC activities and why? (3) What can be improved upon and how? (4) What are unexpected results and their impact? (5) What were the steps taken toward achieving your target for the month?

Feedback was gathered from IPCAs using an open-ended discussion guide to reflect on what worked, review findings from performance data, their perceptions of community response, success stories, challenges encountered, and identify action points for course correction. Action points were documented and shared with all participants and the program manager on an ongoing basis. IPCAs were required to implement action points in the following month and provide feedback in subsequent monthly forums. The pause-and-reflect sessions were iterative in nature, with data on IPCA activities collected routinely, reflected, and acted on for continuous adaptation of implemented activities.

Early IPCA monthly pause-and-reflect sessions and analysis of routine mobilization data revealed some challenges in Lagos State. Data from the implementation of IPC activities carried out individually showed a huge variation in the number of clients referred and the number of redeemed referral slips at CPs and PPMVs' outlets. Most referrals did not translate into clients receiving services at the outlets. A lower number of redeemed referrals were recorded compared to the number of referrals given. Addressing this gap was very important to the success of the project because the overall goal of adopting the IPCA demand generation strategy was to increase contraceptive uptake by increasing the number of clients going to trained CPs and PPMVs for FP services, as evidenced by redeemed referrals. Pause-and-reflect sessions during the IPCA monthly forums helped to identify factors responsible for incomplete referrals, where individuals who were referred failed to show up at the providers' facilities despite indicating interest during contact, and what could be done to improve performance. The responses from IPCAs pointed to similar challenges, which provided the basis for changing the demand generation approach. Further discussions with CPs and PPMVs during providers' quarterly meetings offered further insights that were actionable (Table 1).

**TABLE 1.** Insights on Interpersonal Communication Strategy Gathered Through Responsive Feedback Approach

Learning	Actions Taken	Outcome/Benefits
IPCAs moving singly do not generate as much curiosity and attention of community members as when they move in groups.	IPCAs were grouped together to conduct community awareness.	Increased awareness of IPCAs' activities in communities visited.
One IPCA working in a given community at a time does not have enough time for premobilization activities to the necessary community gatekeepers and local chiefs to create an enabling environment.	IPCAs working as a group are assigned responsibilities that are previously carried out by 1 IPCA.	Group mobilization affords the IPCAs the opportunity to carry out more detailed premobilization activities to necessary gatekeepers.
Nonavailability of trained providers during mobilization of clients to CP/PPMV outlets. Trained providers were not contacted to ascertain times they will be available to provide FP services, so most referred clients were not attended to, leading to missed opportunities for family planning.	There was shared understanding at the pause-and-reflect (break after and-) sessions of the need to schedule mobilization activities in collaboration with CPs and PPMVs to ascertain their availability and nature of other additional health services they provide aside from FP before mobilization.	Stronger relationship between IPCAs and trained providers. Potential clients referred to CPs and PPMVs had access to trained providers.
Number of clients referred does not match the number of clients reporting to the CPs' and PPMVs' outlets.	It was suggested that during mobilization, some IPCAs should provide FP counseling while some IPCAs accompany referred clients to trained providers.	Increased number of clients that completed the referral process.

Abbreviations: CP, community pharmacist; FP, family planning; IPCA, interpersonal communication agent; PPMV, patent and proprietary medicine vendor.

Data from implementation of IPC activities carried out individually showed a lower number of redeemed referrals at CPs and PPMVs' outlets compared to the number of clients referred.

As a result of the pause-and-reflect sessions, a course of action was implemented to improve the number of referrals that were redeemed, namely recruiting new IPCAs. Initially, 4 individual IPCAs were enlisted in Ikorodu for this purpose. Subsequently, 2 additional IPCAs were recruited to increase the total to 6, and they were paired with experienced IPCAs as part of a comprehensive training exercise. During this phase, the collaborative performance among both new and seasoned IPCAs yielded better outcomes compared to their individual efforts. This observation led to the proposed establishment of a group IPC model. The implementation of IPC strategies followed a sequential approach, beginning with individual IPCs and progressing toward a group IPC model. The group IPC strategy encompassed the participation of all 6 IPCAs, including both newly recruited members and those already part of the team, collaborating on outreach activities collectively.

### Program Adjustments and the Results Achieved

Based on these insights and recommendations, the individual IPC strategy was replaced with a group IPC strategy and was initially piloted in 1 local government area before scaling to other focal local government areas in Lagos State. These efforts are considered to have contributed to an increased number of clients who completed the referral process (Figure). The referral success rate showed a noteworthy improvement over time. In quarter 1, the referral success rate stood at 14.3% (95% confidence interval [CI]=13.2, 15.4). However, by quarter 4, there was a significant increase of 16.3% (95% CI=14.5, 18.1), resulting in a referral success rate of 30.6% (95% CI=29.4, 31.9) (Table 2). This indicates a substantial increase in the effectiveness of the group IPC strategy. More clients received FP services due to the presence of trained providers, and contraceptive uptake increased.

**FIGURE:**
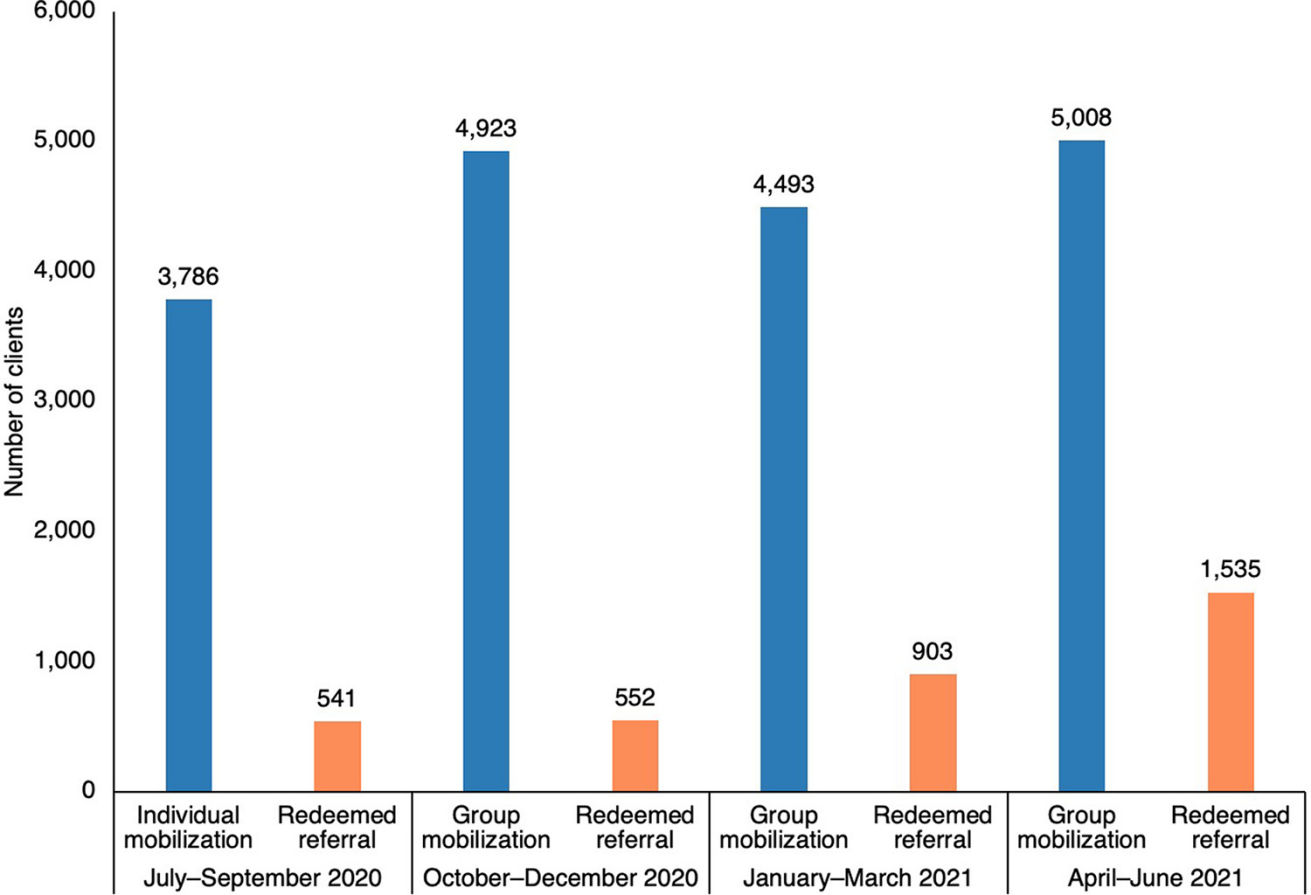
Interpersonal Communication Agent Mobilization Strategy and Redeemed Referrals, July 2020 to June 2021, Ikorodu Local Government Area, Lagos State, Nigeria

**TABLE 2.** Rate of Referral Success per 100 Clients Referred for Each Quarter

Quarter	Clients Referred, No.	Redeemed Referrals, No.	Rate of Referral Success per 100 Clients, % (95% CI)
1	3,786	541	14.3 (13.2, 15.4)
2	4,923	552	11.2 (10.3, 12.1)
3	4,493	903	20.1 (18.9, 21.3)
4	5,008	1,535	30.6 (29.4, 31.9)

Abbreviation: CI, confidence interval.

## WHAT HAVE WE LEARNED?

Applying the same intervention strategy to different geographies without considering the socioeconomic and cultural environment may not provide the best outcome. Although an individual demand generation strategy worked well in Kaduna (Northern Nigeria), it did not work that well in Lagos (Southern Nigeria). As a result, we adapted the IPC strategy to fit the local context of the implementation site. Using a responsive feedback approach helped to systematically gather evidence that informed a timely course correction of the strategy and led to improved contraceptive uptake.

The monthly pause-and-reflect sessions helped IPCAs to see program implementation as a collective responsibility and built their confidence to contribute toward a common goal of improving FP service uptake. There was better coordination among IPCAs that improved the number of community members reached during mobilization activities.

The group IPCA strategy increased the visibility of the IPC agents. Their presence became more noticeable to community members, thereby attracting greater attention and engagement. A more efficient working relationship was developed between trained CPs and PPMVs and IPCAs, thereby reducing missed opportunities for FP of clients referred. The timing of the referrals may have played a significant role in the increased number of referrals. IPCAs engaged in discussions with CPs and PPMVs to determine the periods when they were available, which coincided with community mobilization activities. This synchronization allowed clients to conveniently complete their referrals because the trained providers were present and accessible at the shops and premises during their visits. There was also increased information flow between IPCAs and program managers.

The group IPCA strategy increased the visibility of the IPC agents. Their presence became more noticeable to community members, thereby attracting greater attention and engagement.

Nevertheless, it is important to acknowledge that the group IPC strategy had its disadvantages. CPs and PPMVs faced challenges in effectively managing all the clients referred, including caregivers who were referred for integrated community case management services. This limitation suggested a need for further considerations and potential adjustments to ensure that the increased number of referrals could be adequately handled and attended to by the providers involved. Another drawback of the group IPC strategy was some IPCAs failed to actively contribute to the collective team effort. During the implementation phase, 1 IPCA who displayed traits of inactivity, absenteeism, and a lack of contribution to the team was eventually released from the project. Interestingly, her absence did not have any discernible impact on the overall results, leading to the decision not to replace her with a new IPCA.

Finally, lessons were learned about conducting effective and efficient pause-and-reflect sessions. It is important to incorporate pause-and-reflect sessions into existing activities rather than conduct them as stand-alone activities. Incorporating pause-and-reflect sessions creates a forum for the successful adoption of responsive feedback as part of regular project implementation. Another good practice was conducting the pause-and-reflect sessions with internal project staff and external stakeholders to ensure course corrections were implemented collaboratively and build relationships. The sessions were effective by helping to promote group reflection among IPCAs.^10^

## CONCLUSION

Using a responsive feedback approach by incorporating pause-and-reflect sessions can be an effective way to improve the performance of interventions. This approach provides the opportunity to share lessons learned, generate discussions, provide feedback, and address factors that can have a negative effect on program performance.

## Notes

**Peer Reviewed**

**First published online:** September 15, 2023.
